# The influence of various sample storage conditions and sample bacterial contamination on concentrations of routine biochemical parameters

**DOI:** 10.5937/jomb0-40360

**Published:** 2024-06-15

**Authors:** Amara Gojković, Sandra Vladimirov, Tamara Antonić, Nataša Bogavać-Stanojević, Katarina Novović, Vesna Spasojević-Kalimanovska, Brankica Filipić

**Affiliations:** 1 University of Belgrade, Faculty of Pharmacy, Department of Medical Biochemistry, Belgrade; 2 University of Belgrade, Institute of Molecular Genetics and Genetic Engineering, Belgrade; 3 University of Belgrade, Faculty of Pharmacy, Department of Microbiology and Immunology, Belgrade

**Keywords:** freeze-thaw cycles, bacterial cross-contamination, preservative potassium-fluoride, pre-analytical phase, ciklusi zamrzavanja-odmrzavanja, bakterijska kontaminacija, konzervans kalijum-fluorid, preanalitička faza

## Abstract

**Background:**

The pre-analytical (PA) phase is the most vulnerable phase of the laboratory testing procedure, with critical procedures-collection, handling, sample transport, and time and temperature of sample storage. This study aimed to examine the stability of basic biochemical parameters depending on the samples' storage conditions and the number of freeze-thaw cycles (FTCs). In parallel, the presence of sample bacterial contamination during routine laboratory work was examined.

**Methods:**

Two plasma pools (ethylenediaminetetraacetic acid (EDTA), and sodium-fluoride/potassium oxalate plasma (NaF)) were stored at +4 ˚C/-20 ˚C. Total chole - sterol (TC), glucose, triglycerides (TG), urea, and albumin concentrations were measured using BioSystems reagents (cholesterol oxidase/peroxidase, glucose oxidase/per - oxidase, glycerol phosphate oxidase/peroxidase, urease/ salicylate, and bromcresol green method, respectively) on Ilab 300+. Sample bacterial contamination was determined by 16S rRNA sequence analysis. The expe - riment encompassed a 5 day-period: Day 1-fresh sample, Day 2-1st FTC, Day 3-2nd FTC, Day 4-3rd FTC, Day 5-4th FTC. The appearance of bacteria in two consecutive samples was the experiment's endpoint.

## Abstract (sr)

**Uvod:**

Preanalitička (PA) faza je složen proces koji čine: prikupljanje, rukovanje, transport i skladištenje uzoraka, i predstavlja najznačajniji izvor laboratorijskih grešaka. Cilj ovog istraživanja je bio da se ispita stabilnost osnovnih biohemijskih parametara u zavisnosti od uslova skladištenja uzoraka i broja ciklusa zamrzavanja-odmrzavanja (FTC). Pored toga, ispitivano je prisustvo bakterijske kontaminacije uzoraka tokom rutinskog laboratorijskog rada.

**Metode:**

Dva "pool"-a plazme (etilendiaminotetrasirćetna kiselina (EDTA) i natrijum-fluorid/kalijum oksalat (NaF)) su skladištena na +4 ˚C/-20 ˚C. Koncentracije ukupnog hole sterola (TC), glukoze, triglicerida (TG), uree i albumina su određene korišćenjem BioSystems reagenasa (holesterol oksi daza/peroksidaza, glukoza oksidaza/peroksidaza, glice rol fosfat oksidaza/peroksidaza, ureaza/salicilat, od - nosno bromkrezol zeleno metodama, sukcesivno) na Ilab 300+ analizatoru. Bakterijska kontaminacija uzoraka je potvrđena 16S rRNA sekvencioniranjem. Eksperiment je sproveden tokom 5 uzastopnih dana: 1. dan - svež uzorak, 2. dan - 1. FTC, 3. dan -2. FTC, 4. dan - 3. FTC, 5. dan - 4. FTC. Završnu tačku eksperimenta predstavljala je pojava bakterija u dva uzastopna uzorka.

**Rezultati:**

Tokom 4 FTC koncentracije uree u plazmi se nisu značajno razlikovale. Koncentracija glukoze je bila stabilna u EDTA +4 ˚C i NaF -20 ˚C do 3.FTC (P=0,008, P=0,042, redom). Koncentracije TG su se značajno pro - menile u uzorku EDTA -20 ˚C nakon 1. i 4. FTC-a (P=0,022, P=0,010, redom). U uzorcima NaF plazme nije došlo do bakterijske kontaminacije tokom 4. FTC.

**Zaključak:**

Koncentracije uree i glukoze su bile stabilne tokom trajanja eksperimenta. Promene u koncentracijama lipida nakon FTC prate složene obrasce. Rast bakterija nije primećen u uzorcima NaF plazme, te upotreba ovog anti-koagulansa može biti opravdana u analitičkim procedurama podložnim uticaju mikrobiološke kontaminacije.

## Introduction

Clinical laboratory analysis presents a complex process carried out under controlled conditions, focused on issuing accurate and precise results [1] [2] [3] [4] [5]. It represents an integral part of clinical decision-making. Almost 80% of evidence-based clinical guidelines require laboratory testing [5]. The laboratory testing procedure includes three phases: preanalytical (PA), analytical, and post-analytical [1]. The introduction of state-of-the-art analytical techniques has significantly reduced analytical errors. However, the PA phase still contributes to 46–62.8% of the overall testing errors [1] [2]. There is strong evidence that most PA errors result from manual activities [6]. For most critical procedures in the PA phase (sample collection, handling, transport, and storage), guidelines and quality control measures are not adequate, while compliance with existing guidelines is low [1] [3] [6] [7]. For example, incorrect anticoagulant selection, insufficient sample mixing, and inadequate sample processing represent significant sources of errors. Also, harmonized criteria for sample rejection and guidelines for managing inappropriate samples are necessary [1] [7].

One of the most important measures of laboratory efficiency is the turnaround time (TAT) – the period between sampling and laboratory results delivery. TAT is mostly affected by the PA phase, especially by sample transport and reuse of the primary sample due to verifying previously obtained values or for »add-on« tests [8] [9]. During these procedures, samples stored at room temperature (RT) are susceptible to degradation of labile protein biomarkers, antioxidants, and analytes such as folic acid and vitamin B12. Consequently, concentration change patterns are different and very complex [10].

The microbiome presents a diverse specific community of bacteria, archaea, viruses, and fungi that exist in a particular environment [11]. The ubiquity of environmental microorganisms is a major challenge for maintaining controlled working conditions in health facilities. Microorganisms present in the air, surfaces, equipment, medical devices, or skin and clothing of employees are a potential source of contamination, especially in surgical rooms and laboratories for genetic sequencing [12]. Even though blood and blood derivatives are an ideal medium for the growth of various microorganisms, there is no evidence that possible cross-contamination from the environmental microbiome could occur during sample handling and affect the analyte concentrations [13] [14].

Although many studies were focused on the PA phase, differences in study design protocols have contributed to inhomogeneous, disparate, and inconsistent data, which lead to conflicting con clusions. All of the above indicates that the PA phase still represents an »Iceberg keel« for everyday laboratory practice and requires a more comprehensive view of the PA phaseors [1] [6]. This study aimed to investigate the stability of routine biochemical parameters (RBPs) under different storage conditions, then during freeze-thaw cycles (FTCs) and with the use of different anticoagulants. Furthermore, the goal was to confirm possible bacterial contamination of samples depending on storage conditions, FTCs, and the anticoagulant/preservative used. Thirdly, we investigated whether bacterial contamination was present in the samples, when it occurred, and for which sample types.

## Materials and methods

### Samples, reagents, and analyses

This study used EDTA and sodium-fluoride/potassium oxalate plasma (NaF) pools. Firstly, from each of the 10 healthy volunteers (5 male and 5 female) during yearly medical check-ups in MediGroup Hospital in Belgrade, two 4 mL fully filled vacutainer tubes (BD, New Jersey, U.S) containing corresponding anticoagulants were collected. The participants were selected from the larger group participating in our ongoing study. Each participant signed informed consent, and the study protocol was designed according to the ethical principles of the Declaration of Helsinki. The Ethics Committee of the Faculty of Pharmacy, University of Belgrade, approved the initial study (number 2262/2).

After blood collection, these samples were centrifuged for 10 minutes at 3000 rpm to provide plasma. EDTA plasma samples were combined to make an EDTA pool, whereas the same was done to make a NaF pool. Samples were pooled to minimize interindividual differences in the content of individual components (antioxidants, hormones, etc.), which could affect the results of experiments. Finally, both pools were carefully aliquoted into 2 mL plastic microtubes.

RBPs were analysed using Ilab 300+ biochemical analyser, and commercial analytical reagents (Biosystems, Spain). Metrological characteristics for each investigated parameter are shown through repeatability (within run) and reproducibility (run to run) as coefficients of variation expressed in percentages (CV, %). Repeatability for low and high concentration during 20 repetitions are given respectively: albumin 1.4% and 1.0%, urea 1.6% and 0.8%, triglycerides 1.7% and 0.7%, glucose 1.2% and 0.9%, cholesterol 1.1% and 0.9%. Reproducibility for low and high concentration during 25 repetitions are given respectively: albumin 1.9% and 1.9%, urea 2.4% and 1.3%, triglycerides 2.6% and 1.7%, glucose 2.7% and 1.9%, cholesterol 1.9% and 1.0%. During the experiments, quality control procedures of laboratory work were carried out daily, respecting the Westgard rules. The study was conceptualized to consist of 3 experiments.

### Experiment 1. Investigating the influence of different anticoagulants and FTCs on the stability of RBPs

For this experiment, two 2-mL aliquots of EDTA plasma pool and another two 2-mL aliquots of NaF plasma pool were used. One pair of aliquots (one EDTA and one NaF plasma pool) was stored at +4°C (EDTA +4°C and NaF +4°C), while the other pair was kept at -20°C (EDTA -20°C and NaF -20°C) for 5 consecutive days. Each day, all four aliquots were used for the analysis of RBPs. Samples kept at +4°C were left to RT, while samples stored at -20°C were slowly thawed until they reached RT before running the analyses in triplicates. After the analyses, the sample aliquots were placed back in the refrigerator/freezer, and the above-described procedure was repeated the next day from the same aliquots. The purpose of this experiment was to identify the differences between the concentrations of the examined parameters in EDTA plasma stored at +4°C and -20°C, NaF plasma stored at +4°C and -20°C, but also EDTA +4°C vs NaF +4°C and EDTA -20°C vs NaF -20°C.

### Experiment 2. Analyses of the bacterial growth in differently stored samples

In parallel with Experiment 1, the samples were screened for bacterial contamination on each subsequent day before repeating the analyses. The same amount of aliquot was taken and seeded from each sample on Tryptic Soy Agar (TSA) medium. The inoculated TSA were incubated at 35°C 18-20 h. TSA medium was chosen because it supports the growth of most Gram-positive and -negative bacteria, yeasts, and molds [15].

When bacterial growth was detected on TSA medium after incubation, each colony was subcultivated to TSA and subjected to identification analysis. The steps in identifying the microorganisms present in the sample were as follows: (1) isolation of chromosomal DNA; (2) amplification of the gene for 16S rRNA using PCR and universal primers 16S—Fw (GAATCTTCCACAATGGACG) and 16S—Rev (TGACGGGCGGTGTGTACAAG); (3) PCR sample purification using a commercial kit (Thermo Scientific PCR Purification Kit, Thermo Scientific, Lithuania) and following the manufacturer’s protocol; (4) PCR products were sequenced by Macrogen Sequencing Service (Macrogen Europe, Amsterdam, Netherlands).

Isolated bacteria were identified based on the gene sequence analysis for 16S rRNA. The obtained nucleotide sequences were analysed using the National Center for Biotechnology Information (NCBI) database and The Basic Local Alignment Search Tool (BLAST®) program (https://blast.ncbi.nlm.nih.gov/Blast.cgi).

### Experiment 3. Analysing the influence of bacterial contamination on RBPs

The bacterial contamination of the sample was determined as positive when the bacteria were detected in the sample aliquotes for two consecutive days.

As mentioned above, RBPs were analysed each day, following each FTC. Plasma concentrations of total cholesterol (TC), glucose, triglycerides (TG), urea, and albumin were determined by cholesterol oxidase/peroxidase, glucose oxidase/peroxidase, glycerol phosphate oxidase/peroxidase, urease/salicylate, and bromcresol green method, respectively. RBPs were analysed using Ilab 300+ biochemical analyser and commercial analytical reagents (Biosystems, Spain).

### Statistical analyses

All data were analyzed using IBM® SPSS® Statistics version 21 software. Data distributions were tested using the Shapiro-Wilk test. Normally distributed continuous variables were shown as mean ± standard deviation (SD). Continuous variables were compared by repeated measures analysis of variance (ANOVA) and one-way ANOVA, and the Tukey HSD post hoc test tested between-group differences. We used repeated-measures ANOVA when we examined changes in parameter concentrations during 5 days, stored under the same conditions and collected with the same anticoagulants, and one-way ANOVA when we examined differences in parameter concentrations in biological materials collected with different anticoagulants and stored under different conditions on each day. P values less than 0.05 were considered statistically significant.

## Results

**Figure 1:**
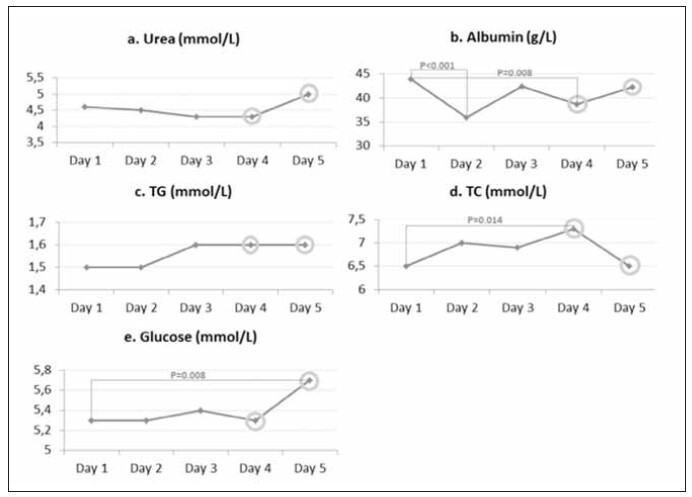
Differences in RBPs measured in EDTA+4°C during five days. Rounded data points indicate the day when bacterial contamination was observed. TG – triglycerides; TC – total cholesterol.

Our results demonstrated that in EDTA+4°C, urea concentrations did not differ between day 1 and the following days (Figure 1a). The same pattern was observed for TG (Figure 1c). Albumin concentrations were significantly lower on days 2 and 4 than on day 1 (Figure 1b). TC concentration was significantly higher only on day 4 compared to day 1 (Figure 1d). Glucose concentration was significantly higher only on day 5 compared to day 1 (Figure 1e). Data were obtained by using repeated-measures ANOVA and, consequently, the Tukey HSD post hoc test. The results of bacterial contamination analysis showed that EDTA+4°C was positive for bacterial contamination (Figure 1). In these aliquotes, *Staphylococcus epidermidis* was identified on day 4, while on day 5, both *Staphylococcus epidermidis* and *Staphylococcus hominis* were identified (rounded data points in Figure 1).

**Figure 2:**
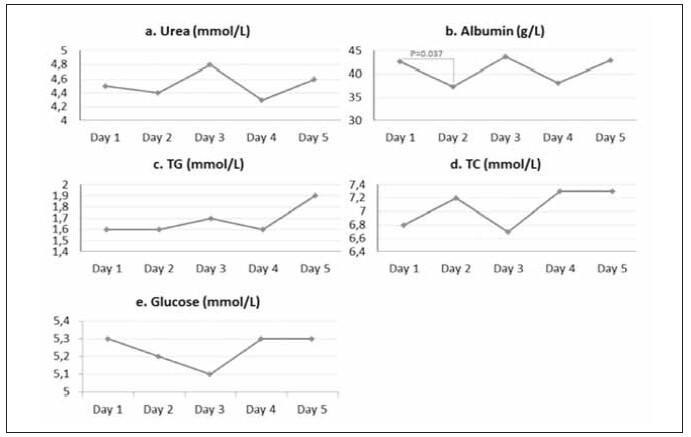
Differences in RBPs measured in NaF +4°C during five days. TG – triglycerides; TC – total cholesterol.

The same analysis was performed in plasma samples obtained using NaF as an anticoagulant (Figure 2). Urea (Figure 2a), TG (Figure 2c), TC (Figure 2d), and glucose (Figure 2e) concentrations did not differ between day 1 and the following days. Albumin concentrations were significantly lower only on day 2 than day 1 (Figure 2b). Data were obtained by using repeated-measures ANOVA and, consequently, the Tukey HSD post hoc test.

In NaF+4°C, no bacterial growth was observed during five days.

**Figure 3:**
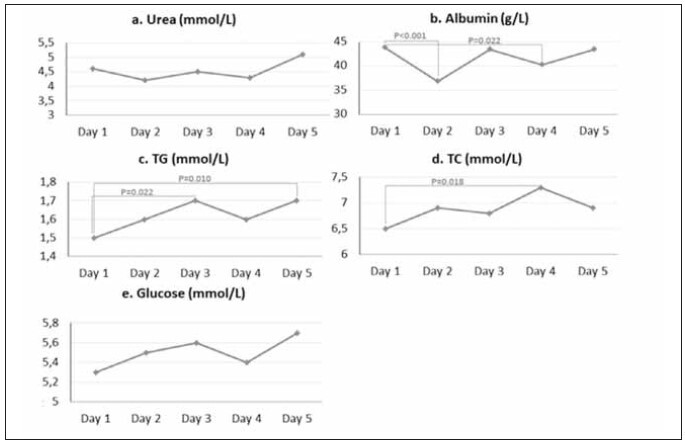
Differences in RBPs measured in EDTA -20°C during five days. TG – triglycerides; TC – total cholesterol.

Urea concentrations did not differ in the EDTA-20°C between day 1 and the following days (Figure 3a). Albumin concentrations were significantly lower on days 2 and 4 compared to day 1 (Figure 3b), while TG concentrations were significantly higher on days 3 and 5 compared to day 1 (Figure 3c). TC concentrations were significantly higher only on day 4 compared to day 1 (Figure 3d), while glucose concentrations were slightly higher only on day 5 compared to day 1 (P=0.056, 5.7±0.35 mmol/L vs 5.3±0.14 mmol/L) (Figure 3e). Data were obtained by using repeated-measures ANOVA and, conse quently, the Tukey HSD post hoc test. EDTA -20°C did not exhibit bacterial growth during the 5-day storage period.

**Figure 4:**
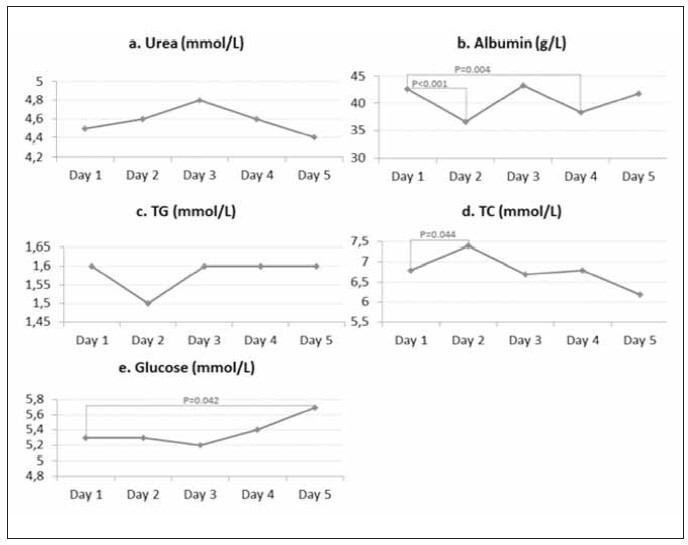
Differences in RBPs measured in NaF -20°C during five days. TG – triglycerides; TC – total cholesterol.

In NaF -20°C during the five days, urea and TG concentrations did not differ between day 1 and the following days (Figure 4a and Figure 4c). Albumin concentrations were significantly lower on days 2 and 4 than on day 1 (Figure 4b). TC concentrations were significantly higher only on day 2 compared to day 1 (Figure 4d). Glucose concentrations were significantly higher only on day 5 compared to day 1 (Figure 4e). Data were obtained using repeated-measures ANOVA and the Tukey HSD post hoc test. In NaF -20°C stored for 5 days, there was no bacterial contamination.

Finally, we performed a one-way ANOVA test with the Tukey HSD post hoc test to identify differences in RBPs depending on the collection method and storage conditions (Table 1).

**Table 1:** Comparison of serological indicators between the two groups (x̄ ±s). TG – triglycerides; TC – total cholesterol. Data are expressed as mean ± standard deviation. Continuous variables were compared using one-way analysis of variance (ANOVA), and the Tukey HSD test tested between-group differences.

Parameter	Day	A	B	C	D	P_A-B_	P_A-C_	P_B-D_
		EDTA<br>plasma, 4°C	EDTA<br>plasma, -20°C	NaF plasma,<br>4°C	NaF plasma,<br>-20°C			
Urea<br>(mmol/L)	1	4.6±0.43	4.6±0.43	4.5±0.4	4.5±0.4	/	/	/
	2	4.5±0.15	4.2±0.08	4.4±0.07	4.6±0.23	0.041	0.368	0.092
	3	4.3±0.03	4.5±0.42	4.8±0.73	4.8±0.38	0.512	0.351	0.424
	4	4.3±0.08	4.3±0.23	4.3±0.05	4.6±0.14	0.820	0.843	0.091
	5	5.0±0.04	5.1±0.01	4.6±0.34	4.4±0.13	0.757	0.081	0.001
Albumin<br>(g/L)	1	43.9±2.29	43.9±2.29	42.7±0.97	42.7±0.97	/	/	/
	2	35.9±0.53	36.8±1.25	37.3±0.67	36.6±1.19	0.282	0.041	0.854
	3	42.4±1.34	43.5±1.01	43.7±3.79	43.3±1.47	0.315	0.622	0.802
	4	38.7±0.87	40.3±1.77	38.2±3.24	38.3±2.19	0.246	0.786	0.298
	5	42.2±3.14	43.5±1.57	42.9±3.29	41.8±0.95	0.561	0.806	0.193
TG (mmol/L)	1	1.5±0.06	1.5±0.06	1.6±0.11	1.6±0.11	/	/	/
	2	1.5±0.11	1.6±0.03	1.6±0.13	1.5±0.09	0.631	0.524	0.826
	3	1.6±0.11	1.7±0.06	1.7±0.15	1.6±0.13	0.230	0.471	0.479
	4	1.6±0.11	1.6±0.09	1.6±0.03	1.6±0.06	0.653	0.390	0.502
	5	1.6±0.08	1.7±0.12	1.9±0.34	1.6±0.23	0.220	0.288	0.455
TC<br>(mmol/L)	1	6.5±0.34	6.5±0.34	6.8±0.67	6.8±0.67	/	/	/
	2	7.0±0.17	6.9±0.27	7.2±0.21	7.4±0.23	0.922	0.260	0.077
	3	6.9±0.49	6.8±0.15	6.7±0.14	6.7±0.24	0.608	0.523	0.556
	4	7.3±0.21	7.3±0.28	7.3±0.3	6.8±0.17	0.990	0.751	0.100
	5	6.5±0.17	6.9±0.48	7.3±1.49	6.2±0.19	0.245	0.414	0.075
Glucose<br>(mmol/L)	1	5.3±0.14	5.3±0.14	5.3±0.21	5.3±0.21	/	/	/
	2	5.3±0.18	5.5±0.18	5.2±0.08	5.3±0.12	0.377	0.364	0.256
	3	5.4±0.08	5.6±0.21	5.1±0.1	5.2±0.13	0.203	0.012	0.057
	4	5.3±0.1	5.4±0.16	5.3±0.08	5.4±0.21	0.560	0.950	0.721
	5	5.7±0.13	5.7±0.35	5.3±0.23	5.7±0.33	0.872	0.109	0.883

Urea concentrations differed significantly on day 2 between EDTA +4°C and EDTA -20°C (Table 1). Also, a significant difference in urea concentrations was observed on day 5 between EDTA -20°C and NaF -20°C samples (Table 1). Albumin concentrations were significantly lower on day 2 when stored at 4°C with EDTA than NaF. Glucose concentrations differed significantly on day 3 between EDTA +4°C and NaF +4°C samples (Table 1). A significant difference was observed on day 3 between EDTA -20°C and NaF -20°C samples (Table 1). All other parameters did not differ according to sample storage conditions and preservatives used.

## Discussion

The quality protocols of laboratory tests should be assured to guarantee that all laboratory procedures are performed correctly, thus providing an accurate and precise result as a diagnostic tool [16]. Steps in the PA phase require better control due to many potential errors, especially during sample storage procedures [1] [2] [3] [4] [5]. Previous studies examined the effect of FTCs on changes in biomarker concentrations [3] [9] [14] [17] [18] [19]. However, we went a step further, providing a holistic approach to this pre-analytical challenge by considering the effects of two different anticoagulants (EDTA, NaF/potassium oxalate), presence of preservative (NaF), storage temperatures (+4°C and -20°C), multiple FTCs, and environmental microorganisms. According to our knowledge, this is the first study to examine the effect of FTC and consequent possible bacterial contamination of samples on RBP concentrations.

It is well-established that FTCs affect biomarker concentration [17] [18] [19]. Although modern analytical techniques, such as chromatographic techniques, can detect small changes in the concentrations of individual markers [17] [18], it should be noted that there is no perfect measurement and that variations in biochemical measurements are an integral part of everyday laboratory work [1] [2] [3] [4] [5]. In this regard, in the material and methods section, we have listed the metrological characteristics of the reagents (repeatability and reproducibility). During the experiments, laboratory work quality control procedures were carried out daily, respecting the Westgard rules. A metabolomic study conducted by Chen et al. [18] examined changes in concentrations of 2790 metabolites after 5 FTCs, and for certain parameters, changes greater than 1.5 times were observed. The results of our study showed that changes in the concentrations of the tested parameters occur during 4 FTCs, except for changes in the urea concentration (Figure 1, Figure 2, Figure 3, Figure 4). It should be noted that we determined the urea concentration in EDTA and NaF plasma samples. Although the manufacturer recommends using heparinized plasma, Taylor et al. [20] state that anticoagulants containing ammonium ions are the only limitation for urea quantification by the BUN method. Urea concentration changes are in a narrow range and are not statistically significant when observing day-to-day FTCs (same anticoagulant and same storage temperature) (Figure 1a–Figure 4a). The results of various studies are inconsistent in terms of changes in urea concentrations after FTCs [14] [19]. In a study conducted by Cuhadar et al. [14], urea concentrations increased significantly during 10 FTCs. The same authors hypothesised, in study limitations, that possible bacterial contamination during FTCs could be one of the unexplored causes of urea concentration change [14]. Our study confirmed the presence of *S. epidermidis* species in a plasma EDTA +4°C, with no changes in urea concentration (Figure 1a). Bacterial species *S. epidermidis, S. epidermidis*, and *S. hominis* were isolated after the 3^th^ and 4^th^ FTC, respectively, in EDTA +4°C (Figure 1). *S. epidermidis* and *S. hominis* are part of human skin microbiota, so laboratory workers who handle samples could be possible sources of contamination [21]. Isolation of the same bacterial species (*S. epidermidis*) from two consequent samples (3rd and 4th FTC in the EDTA +4°C plasma) was considered as cross-contamination of the samples. It defined the end of FTC repetition in our study. Our results confirmed the presence of *S. epidermidis* after the 3^rd^ and 4^th^ FTC in the EDTA 4°C plasma sample (Figure 1). Although the bacterial species *S. epidermidis* can produce the enzyme urease [22], urea concentrations are robust during at least 4 FTCs, and bacterial cross-contamination did not affect the change in their concentration (Figure 1a). On the other hand, the results of a study by Gislefoss et al. [19] showed that the urea concentrations were robust up to 10 repeated FTCs, confirmed by our results (Figure 1a–Figure 4a). However, urea concentrations differed between plasmas with different anticoagulants under different storage conditions. (Table 1). We chose NaF vacutainers due to the antimicrobial effect of NaF [23]. During 4 FTCs, there was no cross-contamination of NaF plasma, indicating that this preservative might be an optimal solution for collecting and handling samples where bacterial contamination must not occur [24].

It is particularly important to examine the influence of FTC on new parameters concentration before their introduction into routine laboratory practice to establish the optimal protocols and reduce the possibility of PA error [17] [18]. Our previous study demonstrated that FTC causes a significant decrease in plasma and serum desmosterol and β-sitosterol concentrations determined by chromatographic technique [17]. Both of these parameters have a similar structure to cholesterol. Possible reasons for the concentration decrease may be the analytes oxidative modifications and a consequent change in their chromatographic properties [17] [25].

In the present study, we observed a significant increase in TC concentrations during FTC 1 (Figure 4d) and FTC 3 (Figure 1d, Figure 3d). However, the increase in TC concentrations during FTCs 1 and 3 was not statistically significant in NaF +4°C (Figure 2d). Our results partly correspond with the results obtained in two different studies. The study by Cuhadar et al. [14] showed that there were no significant changes in TC concentrations after 10 FTCs. In contrast, the results of the Gislefoss et al. [19] showed that there were significant changes in TC concentrations determined in fresh samples. After the first FTC, no significant changes were present through further cycles. We also did not observe differences in TC concentrations (Table 1) after comparing the values obtained in plasma using different anticoagulants under different storage conditions. Although there was an increase in TC concentration after bacterial contamination of EDTA +4°C, there was also a change in concentration in the EDTA -20°C, in which the presence of bacteria was not observed. Concentration changes have unequal complex patterns under different conditions and cannot be attributed to bacterial contamination.

Changes in TG concentrations occurred only in the EDTA -20°C (Figure 3c), while under other conditions, there were no changes in concentrations during 4 FTCs (Figure 1c, Figure 2c, Figure 4c). No significant changes in TG concentrations were observed comparing different storage conditions and anticoagulants after the same number of FTCs. Although they observed significant changes in TG concentration, Cuhadar et al. [14] indicated that the changes did not reach clinical significance, thus not affecting laboratory results interpretation and clinical decisions. Structural changes in lipoproteins during the FTCs can lead to aggregation and changes in the lipid composition caused by the reactivation of enzymes (hydrolysis, synthesis, and lipid transfer between lipoproteins) and non-enzymatic oxidation during storage of samples at RT [17] [26].

Several studies have described the impact of the work environment microbiome on workers [11] [27]. However, questions arise about how the human and environmental microbiome can influence the performance in experimental and routine laboratories [27] [28]. NaF as an anticoagulant is used for the glucose analysis due to glycolysis inhibition and increased sample stability [29]. The antimicrobial activity of NaF has found application in toothpaste [23]. Still, there is no evidence that the NaF amount present in vacutainers is sufficient to prevent the growth and development of microorganisms in plasma. Our study showed no bacterial contamination occurred during 4 FTCs in both plasma samples (+4°C and -20°C) containing NaF (Figure 2, Figure 4). Glucose concentration remained unchanged for 4 FTCs in NaF +4°C (Figure 2) and EDTA -20°C (Figure 3) samples. Glucose concentrations were significantly higher after the 4^th^ FTC in both samples (NaF -20°C and EDTA +4°C) and subsequently cannot be attributed to bacterial contamination present only in the EDTA +4°C (Figure 1e, Figure 4e). On the third day, glucose concentrations showed a significant difference when samples were stored under the same conditions, but with different anticoagulants/preservatives (Table 1). Other studies have shown conflicting results regarding FTC stability of plasma glucose. While glucose was stable for 10 FTCs in some studies, other studies indicated a statistically significant increase in glucose concentration after FTC 1 [14] [19] [30].

The accelerated development of proteomic research and the necessity to discover new biomarkers is required for adequate collection, processing, and storage of many samples and the formation of innovative, modern, and irreplaceable biobanks [10] [31] [32]. Biobanks should ensure proper and professional sample storage in a strictly organized manner, avoiding all known PA errors [31] [32]. Protein stability is questionable due to rapid denaturation, which multiple factors influence. At +4°C, proteins can be temporarily preserved against degradation by preventing protease activity [10]. In our study, we did not determine the concentrations of total proteins. The biuretic reagent is used to determine the content of total proteins in the sample. Using the biouretic reagent, it is possible to determine the concentration of total plasma proteins and proteins present in bacterial contamination [33]. Given that one of the objectives of the study was to examine the bacterial contamination of the samples during FTCs, we decided to measure the concentration of human albumin during the study.

In our study, changes in albumin concentration had a zig-zag pattern (Figure 1b–Figure 4b) that may be due to various effects, including thawing and freezing rates, cryopeptide formation, and protein precipitation [18] [19]. All the above indicates that a comprehensive view of the PA processes is needed to find new errors and prevent existing errors.

We showed that *S. epidermidis* was present in the EDTA plasma after 3 days of storage at +4°C. However, since the RBP concentrations change seen during repeated FTCs and prolonged storage at +4°C exhibit complex patterns, this change could not be attributed to bacteria. The additional limitation of this study was that the cultivation conditions identified bacteria, but not viruses, anaerobic bacteria, and organisms that require special media for cultivation. Also, a limitation of the study is the small sample size. As this is a pioneering study investigating possible bacterial contamination of samples and its influence on the concentration of RBPs, future studies that would investigate this pre-analytical problem, in addition to a larger number of samples, could include different biological materials and a larger number of different analyses.

In summary, urea and glucose concentrations were robust during our study. Through 4 FTCs, there were no changes in urea concentration in samples obtained using EDTA/NaF and stored at +4°C/ -20˚C, even after the appearance of urease-producing bacteria. Glucose concentration was stable until FTC 3. Changes in lipid and albumin concentrations after FTCs are complex and require greater control during the sample-handling process. No cross-contamination of samples obtained using NaF as an anti coagulant occurred during 4 FTCs. This can promote its use as an anticoagulant and preservative, especially in the analytical procedures in which bacterial contamination affects the quality of analysis.

## Dodatak

### Acknowledgment

This study was financially supported by a grant from the Ministry of Education, Science and Technological Development, Serbia (No: 451-03-68/2022-14/200161 and No: 451-03-68/2022-14/ 200042).

### Conflict of interest statement

All the authors declare that they have no conflict of interest in this work.

### List of abbreviations

ANOVA, analysis of variance;<br>FTC, freeze-thaw cycles;<br>NaF, sodium fluoride;<br>PA, pre-analytical phase;<br>TAT, turnaround time;<br>RT, room temperature;<br>RBP, routine biochemical parameters
